# Between protection and flexibility: Uber drivers’ perspectives on regulating platform work in Johannesburg, South Africa

**DOI:** 10.3389/fsoc.2026.1804777

**Published:** 2026-04-07

**Authors:** Percyval Bayane

**Affiliations:** Department of Sociology, University of South Africa, Pretoria, South Africa

**Keywords:** algorithmic management, flexibility, Johannesburg, platform work, precarity, regulation, South Africa, Uber

## Abstract

Debates over the regulation of platform work often hinge on the tension between worker protection and labour flexibility, yet little is known about how platform workers themselves navigate this trade-off in Johannesburg. This study examines Uber drivers’ perspectives on the regulation of platform work in Johannesburg, South Africa, a context marked by high unemployment, migrant precarity, and heightened safety risks. Drawing on semi-structured interviews with 20 Uber drivers, the study uses thematic analysis informed by algorithmic management and precariat theories to interpret how drivers understand, value, and negotiate the conditions of platform labour. The findings reveal a dual position: many drivers support regulation as a means to improve safety, ensure fair earnings, reduce market oversaturation, and gain access to benefits such as pensions and due-process mechanisms for deactivation disputes. Others remain sceptical, expressing concern that formalisation may undermine the flexibility they value, increase deductions from already unpredictable earnings, and introduce additional oversight on top of existing algorithmic control. Across participant accounts, algorithmic opacity, fluctuating operational costs, and income instability emerged as core sources of precarity. The study suggests that drivers’ varied attitudes are rational responses to digital control and ongoing feelings of insecurity, rather than being inconsistent. It concludes that context-sensitive hybrid regulatory models, combining flexibility with enforceable protections, may be better suited to the realities of digital platform labour in South Africa.

## 1 Introduction

Digital platforms have reshaped urban mobility and labour markets worldwide, with ride-hailing services like Uber positioned as flexible, accessible solutions to transport dysfunctions, while simultaneously transferring risks and responsibilities onto workers (De Stefano, 2016; Prassl, 2018; Woodcock and Graham, 2020; Webster and Masikane, 2023). In South Africa, Uber’s launch in Johannesburg catalysed rapid uptake amid high unemployment and uneven public transport quality; yet its growth has unfolded within a contested regulatory environment and volatile socio-economic conditions (Kute, 2017; Henama and Sifolo, 2017; Woodcock and Graham, 2020; Fairwork, 2021; Bayane, 2024). Platform labour’s promise of autonomy and entry-level income sits uneasily alongside chronic insecurity, opaque pricing, and limited access to social protection, tensions that are especially visible in South Africa and in cities facing intense competition with minibus taxis and metered taxis (De Stefano, 2016; Kute, 2017; Anwar and Graham, 2021; Webster and Masikane, 2023; Bayane, 2024).

A central axis of debate concerns the employment classification of platform workers and the implications this holds for regulatory protection. In South Africa, legislation such as Section 200A of the Labour Relations Act and Section 83A of the Basic Conditions of Employment Act introduces rebuttable presumptions to determine whether a worker should be deemed an employee when indicators of control or economic dependence are present (Nemusimbori, 2017). Following a series of driver deactivations, the CCMA initially ruled that Uber drivers qualified as employees for jurisdictional purposes; however, the Labour Court later overturned aspects of this decision, reaffirming the contractual designation of drivers as independent contractors and thereby sustaining a state of legal ambiguity (Nemusimbori, 2017; Newaj, 2023). Comparative rulings highlight the flexibility of platform work categories: the UK Supreme Court recognised Uber drivers as workers, entitling them to minimum wage and holiday pay (Chayya, 2023). These mixed outcomes sustain a regulatory vacuum in which drivers shoulder market risks without formalised benefits or collective voice mechanisms (De Stefano, 2016; Prassl, 2018).

Johannesburg’s transport environment intensifies these dynamics. Persistent conflict between e-hailing drivers and taxi associations, ranging from intimidation to vehicle torchings and violent confrontations, has been widely reported, heightening safety concerns and reinforcing the need for regulatory intervention (Kute, 2017; Simpson, 2022; Simpson, 2023; Bayane, 2026). Earnings are further destabilised by fuel price volatility, with pump prices in South Africa tied to global oil markets, exchange-rate movements, and domestic levies, making operational costs unpredictable for drivers (Fuels Industry Association of South Africa, 2022). Evaluations of platform work in the country consistently document this precarity: ride-hailing platforms frequently fall short on fair pay, no access to formal representation channels, and expose drivers to significant safety risks, amplified by algorithmic opacity and unilateral decision-making (Fairwork, 2021; Webster and Masikane, 2023).

Despite expanding legal, economic, and policy debates on platform labour, drivers’ own perspectives on regulation remain underexplored in the South African context. Existing scholarship has highlighted how algorithmic systems structure labour processes through information asymmetries, performance metrics, and digital control (Roseblant and Stark, 2016; Kute, 2017; Geitung, 2017; Chinguno, 2019), while broader theorisation frames platform work as characteristic of contemporary precarity (Kute, 2017; De Stefano, 2016; Bayane, 2024). However, empirical accounts of how drivers themselves negotiate the trade-off between protection (benefits, safety, earnings stability) and flexibility remain limited, particularly in contexts marked by legal ambiguity, competition-driven conflict, and cost volatility (Woodcock and Graham, 2020; Anwar and Graham, 2021; Webster and Masikane, 2023). This article addresses that gap and is guided by the primary research question: How do Uber drivers perceive the regulation of platform work, and what forms of regulation do they deem appropriate?

## 2 Literature review

A growing body of scholarship shows that platform firms pitch flexibility and autonomy to workers while architecting business models that externalise risk and circumvent employment protections through contractor designations (De Stefano, 2016; Kute, 2017; Prassl, 2018; Woodcock and Graham, 2020; Anwar and Graham, 2021). Digital platform labour expands amidst high unemployment and constrained formal opportunities, but workers face volatile demand, opaque pricing, and thin safety nets, conditions that sharpen tensions between protection and flexibility (De Stefano, 2016; Anwar and Graham, 2021). Syntheses of gig-economy regulation argue that context-sensitive approaches are required, balancing innovation and access with minimum labour standards and enforceable protections (De Stefano, 2016; Woodcock and Graham, 2020).

Debates on regulation hinge on worker classification, whether platform workers are employees, workers, or independent contractors, because classification determines access to rights. Comparative work traces divergent court outcomes: the UK Supreme Court recognised Uber drivers as workers entitled to minimum wage and holiday pay, while South African courts have tended to uphold independent contracting despite CCMA findings to the contrary (Chayya, 2023; Newaj, 2023). The overturning of CCMA findings sustained legal ambiguity, enabling platforms to allocate market risks to drivers while avoiding obligations related to collective voice and benefits (De Stefano, 2016; Prassl, 2018). However, the classification debate is increasingly viewed as insufficient for addressing the organisational and institutional challenges of platform labour. Maric et al. (2025) advance this discussion by proposing functional platform-mediated work systems, arguing that regulation must redesign organisational elements, such as transparency, rule clarity, and due-process safeguards, rather than fixate solely on employment status labels.

Research on algorithmic management highlights how platforms govern labour through allocation algorithms, ratings, surge prompts, incentive quests, and automated deactivation thresholds, producing compliance without traditional supervision (Roseblant and Stark, 2016; Chinguno, 2019; Kellogg et al., 2020; Cameron, 2024). Uber creates an environment of information asymmetries, where drivers have limited knowledge about demand trends, pricing strategies, and performance metrics. This lack of information leads to dependence and uncertainty for drivers, despite the platform’s narrative promoting entrepreneurship and freedom (Roseblant and Stark, 2016; Rosenblat, 2018). Scholars conceptualise workers’ flexibility as contingent autonomy: drivers can choose when to log in, but earnings and penalties are structured by code, market saturation, and opaque rules (Chinguno, 2019; Kellogg et al., 2020; Woodcock and Graham, 2020). Recent research on organisations shows that digital platform governance creates a sense of consent through limited options available to users (Cameron, 2024). Karunakaran (2024) shows that digitally mediated systems can obscure accountability for frontline professionals, creating ambiguity about who is responsible for decisions, actions, and outcomes in these environments. This situation highlights the necessity for clear accountability mechanisms in platform work. To address this issue, a model of multisided accountability is recommended, as it combines the input of workers from the bottom up with legal and regulatory protections from the top down, aiming to rebalance power dynamics in platform work (Rahman et al., 2024).

Platform labour is marked by chronic income instability, limited social protection, and blurred employment relations, intensifying insecurity for workers who shoulder operational costs and liabilities (De Stefano, 2016; Kute, 2017; Anwar and Graham, 2021; Webster and Masikane, 2023; Bayane, 2024). In South Africa, independent assessments document low effective pay after costs and waiting time, limited channels for representation, and heightened safety risks for e-hailing and delivery workers (Fairwork, 2021). Research further highlights persistent shortfalls in ride-hailing platforms, especially around due process, transparency of algorithmic management, and the absence of robust worker representation (Heeks et al., 2021; Fairwork, 2021). Johannesburg presents particular safety concerns due to recurrent conflict between e-hailing drivers and taxi associations, including intimidation, assaults, and vehicle torchings, making regulation salient for conflict mitigation and driver protection (Simpson, 2023; Bayane, 2026).

E-hailing earnings are highly sensitive to fuel costs and platform pricing. In South Africa, pump prices are regulated and tied to international oil benchmarks, shipping, exchange-rate movements, and domestic levies, generating month-to-month volatility for drivers (Fuels Industry Association of South Africa, 2022). Studies of passenger transport further link fuel prices, unemployment, and exchange-rate fluctuations to shifts in mobility and demand, underscoring how macro-economic uncertainty feeds back into platform incomes (Ntshingila, 2022). Within this landscape, algorithmic opacity, dynamic pricing, incentive schemes, and fare-sharing rules not transparent to drivers compound financial unpredictability (Roseblant and Stark, 2016; Fairwork, 2021).

Scholars increasingly advocate hybrid/partial regulation that preserves genuine scheduling discretion while instituting minimum earnings floors, social insurance contributions, safety protocols, and transparent due process for deactivation (De Stefano, 2016; Prassl, 2018). This aligns with reform agendas calling for collective representation and platform accountability, emphasising that atomised workers require institutional channels to negotiate conditions, an area where South African platforms have scored poorly (Fairwork, 2021; Heeks et al., 2021). Martindale et al. (2024) also report that platform workers strongly support labour rights, representation, and codetermination while still valuing autonomy, an empirical pattern that resonates with drivers’ preferences for protections without the loss of flexibility.

## 3 Theoretical framework: algorithmic management and precariat theories

This study draws on algorithmic management theory and precariat theory to explain Uber drivers’ uncertain attitudes towards the regulation of platform work, seeking protection while fearing the loss of flexibility. Algorithmic management conceptualises how digital platforms coordinate, monitor, and discipline labour through data-driven systems rather than traditional, face-to-face supervision (Kellogg et al., 2020; Roseblant and Stark, 2016; Christin, 2017; Chinguno, 2019; Webster and Masikane, 2023; Stark and Vanden Broeck, 2024). These dynamics align with recent theoretical developments that conceptualise algorithmic management as a distinct organisational form grounded in the co-optation of users and data flows into managerial functions (Stark and Vanden Broeck, 2024). In ride-hailing, allocation algorithms, dynamic pricing, customer ratings, and automated deactivation thresholds operate as mechanisms of direction, evaluation, and discipline, shaping when, where, and under what conditions drivers can work while maintaining a narrative of independence (Kellogg et al., 2020; Roseblant and Stark, 2016; Chinguno, 2019). These control processes mirror evidence that algorithmic systems tighten oversight and limit genuine discretion (Cameron, 2024). This governance is opaque and asymmetric: platforms possess extensive knowledge of demand flows, price setting, and performance metrics, generating information asymmetries that condition workers’ strategies and earnings opportunities (Rosenblat, 2018; Woodcock and Graham, 2020; Cameron, 2024). Such opacity reflects systemic issues in algorithmic management wherein datafied organisational processes reinforce power differentials and obscure managerial decision rules (Burrell and Fourcade, 2021).

Within this frame, drivers’ commending of flexibility can be understood as contingent autonomy: while latitude over log-in times exists, revenue streams, incentives, and penalties are structured by code and metrics that delimit practical choices. The platform’s algorithmic nudges, such as surge prompts, acceptance-rate signals, and incentive quests, cultivate responsiveness while externalising operational risk onto workers, generating a paradox of perceived freedom under infrastructural control (Roseblant and Stark, 2016; Chinguno, 2019). Research on the principles of algorithmic management similarly emphasises how platforms combine co-optation, continuous data capture, and rule-based governance to orchestrate labour in ways that appear flexible yet systematically constrain autonomy (Stark and Vanden Broeck, 2024). This is a contradictory unity of autonomy and constraint, whereby algorithmic systems enhance coordination and efficiency while intensifying surveillance, bias, and dehumanisation (Burrell and Fourcade, 2021). Therefore, this theory helps explain the ambivalence of Uber drivers’ perceptions about regulation: if algorithmic systems already orchestrate work, formal regulation becomes simultaneously attractive, promising protections against volatility and arbitrariness, and anxiety-provoking for fear it may curtail the limited scheduling discretion drivers value (Kellogg et al., 2020; Woodcock and Graham, 2020).

Precariat theory situates these micro-dynamics within broader political-economic transformations of work marked by chronic insecurity, fragmented employment relations, and attenuated social protection (Standing, 2011). Platform labour intensifies these conditions by shifting costs (vehicles, fuel, maintenance), liabilities (safety, insurance), and market risks (demand fluctuations, oversupply of drivers) onto workers while contesting their status as employees, thereby limiting entitlements to benefits and collective bargaining (De Stefano, 2016; Prassl, 2018; Webster and Masikane, 2023; Masikane and Webster, 2025). In contexts like Johannesburg, characterised by high unemployment, migrant labour, and uneven enforcement, these pressures are intensified, producing a structurally precarious workforce for whom gig work is both an entry point to income and a site of heightened vulnerability (Anwar and Graham, 2021; Woodcock and Graham, 2020; Bayane, 2024).

From this perspective, drivers’ calls for regulation are structurally rational demands for predictability, recognition, and protection, minimum earnings floors, contributions to social insurance, safety provisions, and due-process mechanisms, rather than merely preferences for better platform policies (De Stefano, 2016; Prassl, 2018). At the same time, the desire to retain temporal autonomy reflects how precarity is not only economic but also temporal; workers value control over hours amid unstable income streams (Anwar and Graham, 2021; Bayane, 2024). Precariat lens and algorithmic management clarify why many drivers gravitate towards hybrid or partial regulation: arrangements that preserve real scheduling discretion while instituting protections to mitigate volatility, misclassification harms, and the unilateral power embedded in platform infrastructures (De Stefano, 2016; Woodcock and Graham, 2020).

## 4 Methodology

This study was guided by the qualitative research design as it aimed to gain an in-depth understanding of Uber drivers’ perceptions of whether Uber driving should be regulated or not. According to Babbie (2021), qualitative research is focused on understanding the social world and studied phenomenon based on participants’ views and experiences. Bless et al. (2013) added that the qualitative design enables researchers to thoroughly examine human experiences and actions of what is being studied. Similarly, the qualitative research design enabled me to gain a deeper understanding of Uber drivers’ perceptions on the issue of regulating Uber driving, as I personally interacted and interviewed drivers matching the characteristics of the study, which was being an Uber driver operating in Johannesburg.

In South Africa, Uber first launched in Johannesburg in 2013 before expanding to other metropolitan cities (Henama and Sifolo, 2017; Lakemann and Lay, 2019; Wilson, 2013). Uber introduced its transport services in Johannesburg in 2013, and the services were welcomed by many people for accessibility and efficiency as compared to their counterparts—traditional minibus taxis and buses (Wilson, 2013). As the country’s economic hub, Johannesburg attracts diverse workers and passengers, and ride-hailing has become an important livelihood strategy (Fairwork, 2021; Webster, 2020; Wilmans and Rashied, 2021). Johannesburg was therefore selected not only for its early adoption and scale, but because its operating conditions make it a critical case for analysing drivers’ regulatory preferences: a large and continually expanding driver pool intensifies market saturation; recurrent safety risks and taxi-association confrontations make institutional recognition and police responsiveness central to protection; and algorithmically mediated pricing and allocation, against volatile fuel costs, amplify earnings opacity and income instability (Simpson, 2023). These interacting conditions map directly onto algorithmic management and precarity, allowing the study to observe why hybrid or partial regulation is preferred while still preserving the contingent autonomy drivers rely on. Therefore, Johannesburg was a suitable research site to investigate Uber drivers’ perceptions of regulating Uber driving.

Uber drivers specifically operating in Johannesburg were the key participants of this study. Non-probability’s convenience sampling technique was used to access and select participants of the study. Convenience sampling constitutes the researcher using their knowledge to approach and select individuals matching the characteristics of the study (Etikan et al., 2015; Babbie, 2021). In the same manner, I visited several malls and shopping centres around Johannesburg to find Uber drivers. I knew that finding Uber drivers in these areas would be easier because people need and will use Uber services to travel from and to the malls or shopping centres. Upon arrival in malls and shopping centres, I specifically went to the parking and asked security guards where I would find Uber drivers, and I was referred to their parking spots. I therefore personally approached cars and asked if they were working for Uber, and upon their confirmation, I requested prospective participants matching the characteristics to participate in the study, to be interviewed. To match the characteristics of the study, drivers had to work for Uber or use the Uber app and operate as a driver, and significantly in Johannesburg. While most Uber drivers agreed to be part of the study, others rejected, citing that being interviewed would take up their time as they were there to make money. Nonetheless, at the end of fieldwork, I managed to interview 20 Uber drivers. Given the use of convenience sampling, the findings should be understood as indicative rather than statistically representative, offering analytical insights into patterns within the Johannesburg ride-hailing workforce rather than claims of generalisable prevalence.

The Uber drivers interviewed in this study were between 25 and 49 years old, with most in their early 30s, and had been driving on the platform or with experience spanning from 6 months to 6 years. Educational backgrounds varied, although the majority had not completed matric, while a smaller group held Grade 12 or post-school qualifications such as N6 engineering certificates, diplomas, and bachelor’s degrees. This reflects both limited formal educational attainment for some participants and clear patterns of underemployment among those with specialised or tertiary training who were unable to find work aligned with their qualifications. Participants came from a range of provinces/countries, demonstrating Johannesburg’s pull as an economic centre. Drivers had migrated from Limpopo, Mpumalanga, KwaZulu-Natal, North West, and Zimbabwe, with internal and cross-border migration shaping how they navigated livelihood pressures and responsibilities. These diverse age, education, and migration profiles reveal a heterogeneous workforce for whom ride-hailing serves as both an entry-level survival strategy and, for many, a long-term primary livelihood within Johannesburg’s competitive and high-risk mobility sector.

In total, 20 semi-structured interviews were conducted with Uber drivers, each lasting approximately 55 min. All participants were provided with an information sheet and signed informed-consent forms. To ensure confidentiality, pseudonyms were assigned to all drivers, and identifiable details were removed from transcripts. Audio recordings and digital files were stored in encrypted folders accessible only to the researcher. Semi-structured interviews were conducted with all the participants. Interviews were conducted at Uber drivers’ cars, as they cited being comfortable with interviews conducted in their vehicles. Interviews were conducted while drivers were waiting for ride requests, allowing participants to take part without disrupting their work schedules. All the interviews were using vernacular languages such as IsiZulu, Xitsonga, and Sesotho, with partial English, and this was to ensure that Uber drivers narrate their views in a comfortable language. As many as interviews were conducted in vernacular languages; all interviews were verbatim transcribed to English through listening to the recordings, as interviews were recorded, and I also used fieldnotes to ensure that participants’ perceptions were fully captured.

Thematic content analysis was adopted to analyse and present the data, following the steps of transcribing, coding, and developing themes (Rosenthal, 2016). First, as all interviews were audio-recorded, I began data analysis by transcribing them verbatim and checking each transcript against the audio to ensure accuracy. Second, I undertook open coding through repeated readings of each transcript, attending to similarities and differences in participants’ accounts of safety, autonomy, earnings, platform governance, and regulatory expectations. Initial codes included, for example, can log in/out anytime, deductions may increase, fear of taxi violence/attacks, static fares despite rising costs, and deactivation without warning. These codes were subsequently grouped into categories and refined into broader themes and sub-themes. For instance, the codes such as fear of taxi violence/attacks and deactivation without warning formed part of the theme regulation as protection, while codes such as can log in/out anytime and deductions may increase contributed to the theme regulation as threat. Additionally, codes including government must regulate safety, and partial regulation with optional benefits informed the theme hybrid/partial regulation, which captured drivers’ proposals for combined state- and platform-led approaches. Throughout the analysis, I maintained analytic memos to document coding decisions and theme revisions and also used colour-coding to organise categories (Rosenthal, 2016). Interpretation involved examining how these themes collectively addressed the primary research question.

Interviewing Uber drivers was not easy because my position as a researcher and academic made drivers reluctant to relate to me at first. Uber drivers, before interviews, asked me what I do for a living, and upon my response, others refused to participate in the study, translating that the position of being a researcher and academic impacted the research process, especially during data collection (Bayane, 2025). However, upon realising the impact of my positionalities, I decided to informally introduce myself and jokingly requested to work with them, which assisted them to accept or relate with me, and end up agreeing to participate in the study. Reflexivity, which entails researchers reflecting on their possible impact in the research, is important (Bayane, 2025). Ethical clearance for this study was obtained at University of Johannesburg Faculty of Humanities Research Ethics Committee.

## 5 Findings: between protection and flexibility in Uber driving

This section presents findings from 20 semi-structured interviews with Uber drivers operating in Johannesburg. The findings reveal a complex dilemma where drivers are caught between the need for protection and the potential drawbacks of regulation. Uber drivers articulate an ongoing dilemma: regulation is desired to secure protection and benefits, yet feared for its potential to erode flexibility and reduce take-home earnings. Participants emphasise safety, income volatility, algorithmic opacity, and market oversupply as core pressures shaping their views.

### 5.1 Regulation as protection

Regulation surfaces in drivers’ narratives not as a blanket demand for state control, but as a pragmatic antidote to three frictions produced by platformisation: safety deficits, market disorder, and earnings opacity. Many Uber drivers therefore support regulation to enhance safety, improve working conditions, and secure access to benefits (e.g., pensions) alongside fair earnings. For example, when asked whether Uber should be regulated, Jackson stated:

*“Yes, it must be regulated because it will firstly help to ensure the safety of Uber drivers. Uber drivers would be taken seriously when we report a case of being attacked to the police, and it would be known who Uber drivers are because our work would be regulated. Secondly, the regulation of Uber driving would limit the clashes between Uber drivers and taxi drivers, as we would know who operates where and avoid stepping on each other’s toes. Lastly, regulating Uber driving would also help clients to freely choose which transport to use without fear that drivers might be attacked by taxi drivers during a trip.”* (Jackson, Uber driver).

Jackson’s account highlights three interlinked rationales: driver safety and recognition by authorities, regulatory clarity to de-escalate conflict with traditional taxi operators, and consumer confidence that trips can proceed without intimidation. Given frequent reports of confrontations and attacks in Johannesburg, Uber drivers argue that formal rules would both recognise Uber work and structure responses to risk, through clearer operating norms, territorial guidance, and improved police responsiveness. Several participants echoed the link between regulation and recognition. For example, Jack explained:

*“When something happens on the road, police act like they do not know us, so regulation would show them we are drivers and trying to make money to survive as everyone else.”* (Jack, Uber driver).

Such a response points to the desire for institutional visibility as a form of basic protection. This also reflects core mechanisms of algorithmic management whereby platforms externalise operational risks, such as safety, demand fluctuations, and price instability, onto workers while retaining control over information and coordination systems. Drivers’ emphasis on safety demonstrates how, in the absence of platform-provided protection, physical risk becomes an extension of digital precarity. Additionally, Jackson’s emphasis on safety and visibility therefore highlights how algorithmic labour systems generate both economic and spatial insecurity when not matched by institutional safeguards.

Beyond safety, drivers also link regulation to earnings stability. A recurring concern is oversupply on the platform, which dilutes trip volumes and intensifies competition. Gerald explained:

*“Uber as a company has many drivers, meaning they are making more money than we drivers. So, regulating Uber driving might help from their side to have a limited number of drivers so that we can make enough money to survive.”* (Gerald, Uber driver).

The concern over an excess supply of drivers is a common challenge in the gig economy, where digital platforms continuously accept new workers without considering market saturation. As such, with more drivers competing for the same pool of passengers, earnings become highly inconsistent, pushing some to work longer hours to make ends meet. Uber drivers in this study thus shared that regulation could help limit or control driver intake numbers, preventing market oversaturation and ensuring a more sustainable income for those who rely on Uber driving as a primary source of livelihood. Similarly, Ntsako expressed frustration over Uber’s pricing model, stating:

*“Petrol prices and other things are increasing, but Uber does not increase their prices for drivers to at least earn more.”* (Ntsako, Uber driver).

Ntsako’s frustration captures the mismatch between rising operational costs and opaque, platform-set fares, leaving drivers to absorb inflation without wage indexation. This issue was echoed by many Uber drivers in this study, for instance, Paul said: “Fuel goes up every month, but the rate never changes. Maybe regulation will force them to adjust it.” In this context, regulation is perceived not simply as institutional oversight but as a mechanism that can assist with earnings indexation, price transparency, and protection against algorithmic opacity. Uber drivers who favour regulation envision practical protections, recognition, and safety protocols, measures to address market oversupply, and earnings guarantees that respond to cost inflation. For them, a regulated environment promises both legibility (who may operate, where, and under what safeguards) and stability (predictable earnings and benefits), without which Uber work is experienced as precarious and exposed. Therefore, regulation is not imagined as state intrusion but as a corrective to the instability and ambiguity produced by digital platform-controlled markets.

### 5.2 Regulation as threat

While many drivers endorse regulation for protection, a significant minority frame it as a threat to what they value most about digital platform work: temporal autonomy and control over take-home income. For these participants, formalisation risks adding layers of oversight and new deductions to a job already structured by in-app rules, resulting in a perceived loss of autonomy. Mahlatsi characterises Uber driving as self-employment chosen precisely for the freedom to opt in and out without penalty:

*“Uber is not formal work but self-employment. As individuals, we solely decide to join Uber and when to leave, and there is no penalty or person forcing anyone.”* (Mahlatsi, Uber driver).

Mahlatsi’s account highlights how drivers equate employment with fixed schedules and managerial supervision and thus read regulation as a potential re-imposition of clock time. This helps explain why some resist state-style formalisation even as they acknowledge risks: autonomy is experienced as the compensating benefit of an otherwise uncertain job. Another driver made the same point in pragmatic terms: “Some days I work four hours, other days fourteen. If they fix shifts, I cannot balance family and the late-night surges.” (Enoch, Uber driver). The fear of losing flexibility through regulation indicates that algorithmic management has already configured autonomy as a scarce resource that drivers protect. Flexibility thus functions as a form of contingent autonomy that drivers actively use to hedge income volatility rather than as a simple preference for informality.

Concerns about deductions and a potential decline in net income are significant factors. Many people are worried that mandatory contributions for benefits could reduce their disposable income. John specifically expressed his worries regarding the deductions for these benefits:

*“Formalising Uber would make things worse as drivers’ earnings might be deducted more for benefits, and that would mean we will have less money.”* (John, Uber driver).

For John and others, the issue is not about the benefits, but the timing and distribution of costs within a financially unclear earnings model that is already affected by fare uncertainty, commission deductions, and fluctuating fuel prices. As Reginald put it, “They will say it is for benefits, but we will not even know how much they take from each trip.” (Reginald, Uber driver) In this context, any additional deductions are seen as further risks imposed on drivers. The objection is thus less a rejection of protection per se than a response to algorithmic opacity: without transparent pricing and commissions, any new contribution feels like a net loss. Consequently, their opposition to regulation stems from practical concerns. Drivers are not against labour protections, but they are weighing the trade-offs. If regulations lead to less scheduling flexibility and reduced take-home pay, drivers will resist them. However, if regulations provide real flexibility while ensuring a stable income, such as through clear due-process rules without mandatory long shifts or benefits that do not diminish their immediate earnings, this resistance may lessen. Resistance to regulation can be seen as a logical response from drivers who feel that their only control lies in deciding when and how long they work. This reaction arises in a system where other important aspects, such as pricing, allocation of rides, and deactivation, are managed centrally by the platform. This ongoing tension of wanting protection without paternalism paves the way for the hybrid solutions many drivers propose.

### 5.3 Government regulation oversight with hybrid/partial protections

A portion of drivers view the government as the most appropriate regulator, primarily because Uber operates as a major transport actor whose earnings, market influence, and safety implications extend beyond private contractual arrangements. Mike argued:

*“The government should ensure that Uber is regulated because the app is making a lot of money, which should be taxed and accounted for.”* (Mike, Uber driver).

For drivers such as Mike, regulation is not only about worker protection, but also about ensuring that Uber participates in a formal legal and tax framework. Given that Uber positions itself as an intermediary rather than an employer, this group expresses concern that the absence of state oversight enables corporate opacity, weak accountability, and uneven enforcement of safety standards. State-led regulation is therefore imagined as a route to institutional legitimacy, transparency, and stronger protection. As another driver, Joe, noted, “Only the government can tell the police and taxis how to treat us. Uber cannot stop those fights on the road.” (Joe, Uber driver). This highlights that Uber drivers differentiate between the forms of authority required: the state is expected to govern territorial order and public safety, whereas Uber is expected to govern platform rules and earnings transparency.

By contrast, other drivers argue that Uber itself should regulate aspects of the work, citing the practical reality that the platform already sets fares, allocates trips, and manages performance. Vukosi pointed out:

*“I think Uber itself should ensure that driving is regulated because, as drivers, we do not know each other but only see one another on the road.”* (Vukosi, Uber driver).

This reflects both the proximity of platform governance and the fragmentation of the workforce, which makes collective mobilisation difficult. Regulation, in this framing, is imagined as internal reform, clearer deactivation processes, better driver support, and pricing transparency, implemented by the platform that already governs the labour process. Several participants also emphasised that this internal reform should include due-process mechanisms: “If Uber blocks you, they must tell you why and give you a chance to appeal in the app.” (Sipho, Uber driver). This highlights that drivers view Uber as the appropriate regulator for procedural fairness in areas where algorithmic management already governs work allocation and discipline.

Nevertheless, many Uber drivers advocated for a partial regulation that would grant benefits while preserving flexibility. James said:

*“Uber should be partially regulated so that Uber drivers can have access to benefits, but working conditions should remain – drivers should be allowed to decide when and how to work but pay for benefits.”* (James, Uber driver).

Drivers envision safeguards such as minimum earnings thresholds, insurance or pension options, and transparent grievance/deactivation mechanisms, without imposing fixed shifts or removing the flexibility they value. Another participant stressed that such protections should remain optional: “Some of us want benefits, some do not. Give us a choice so we do not lose our freedom.” (Solomon, Uber driver). Hybrid regulation represents a negotiated response to the contradictory unity of autonomy and constraint embedded in algorithmic management. Drivers seek institutional protections against precarity while preserving the contingent autonomy that enables them to navigate Johannesburg’s volatile labour market. Their proposals effectively allocate regulatory functions across institutions: the state for safety, legitimacy, and market order; the platform for transparency, due process, and operational clarity. This view resonates with calls in the literature for hybrid approaches that balance worker protection with platform flexibility. The findings reveal mixed views on regulatory authority and design. Some drivers emphasise state oversight to secure tax compliance, legitimacy, and safety enforcement; others prefer platform-led responsibility for operational transparency and due process; many converge on hybrid/partial models that deliver baseline protections while preserving temporal autonomy.

## 6 Discussion

This study examined Johannesburg Uber drivers’ views on regulating platform work, revealing a patterned ambivalence: drivers seek protection, safety, due process, earnings stability, and access to benefits, yet fear losing flexibility and net income through formalisation. These seemingly contradictory positions are rational responses to algorithmically mediated control and chronic insecurity within Johannesburg’s volatile transport ecology (Roseblant and Stark, 2016; De Stefano, 2016; Kellogg et al., 2020; Woodcock and Graham, 2020). Drivers’ accounts of opaque pricing, unilateral deactivations, surge prompts, ratings pressure, and oversupply reflect central dynamics of algorithmic management, where information asymmetries and behavioural nudges reshape autonomy as contingent rather than absolute (Roseblant and Stark, 2016; Rosenblat, 2018; Kellogg et al., 2020). Flexibility thus functions as a compensating benefit within an otherwise uncertain labour process, while calls for regulation highlight the need for predictability, such as earnings floors indexed to fuel costs, public recognition, and procedural transparency. The findings further show that regulatory preferences are shaped not only by economic insecurity but by persistent taxi-association violence and poor police responsiveness, making safety a core dimension of platform precarity in this setting.

These findings reinforce and also extend developments in algorithmic management theory that conceptualise platforms as rule-based, data-driven organisational systems whose governance structures are co-constructed with local social conditions (Stark and Vanden Broeck, 2024). In Johannesburg, algorithmic oversight intersects with high-crime environments, taxi-industry hostility, and limited institutional enforcement, intensifying the impact of information asymmetries and automated control. This context demonstrates that algorithmic management does not operate as a digital phenomenon but is shaped by the structural vulnerabilities present in Johannesburg (Burrell and Fourcade, 2021). The findings also theoretically add to the nuanced understanding of the concept of contingent autonomy. While flexibility is algorithmically constrained through metrics, nudges, and ratings systems, drivers in Johannesburg value autonomy as a means of navigating unstable earnings, threats of violence, and volatile demand. This shows that autonomy becomes a practical coping mechanism in precarious socio-economic contexts, revealing a tension within algorithmic management. Flexibility is simultaneously structured by the platform and strategically used by workers to manage insecurities. Drivers’ differentiated expectations of the state and the platform, assigning the state responsibility for safety and market order, and Uber responsibility for pricing transparency and due-process mechanisms, demonstrate a practical allocation of regulatory functions that extends existing models of hybrid governance.

Precariat theory also highlights how digital platform labour externalises costs and risks, blurs employment relations, and restricts access to social protection, and these dynamics are intensified in Johannesburg by high unemployment, migrant precarity, and persistent safety threats (Standing, 2011; De Stefano, 2016; Anwar and Graham, 2021; Fairwork, 2021; Webster and Masikane, 2023; Bayane, 2024; Masikane and Webster, 2025; Bayane, 2026). As such, drivers’ support for hybrid regulatory models, flexibility combined with enforceable protections, emerges as structurally coherent (Prassl, 2018; Woodcock and Graham, 2020). The findings further extend precariat theory by demonstrating that precarity is simultaneously economic, temporal, algorithmic, and spatial. Drivers navigate volatile fuel costs, opaque wage-setting, sudden deactivations, market oversaturation, and physical threats from taxi associations. These overlapping insecurities suggest that platform precarity in Johannesburg operates at multiple scales (Standing, 2011; Bayane, 2024). Therefore, algorithmic management and precarity are mutually reinforcing; algorithmic systems structure access to income and work opportunities, while broader institutional and spatial vulnerabilities shape how drivers experience and interpret this control.

Safety concerns remain fundamental. Reports of intimidation, attacks, and vehicle torching at the interface with taxi associations render safety and territorial clarity central to drivers’ regulatory demands (Simpson, 2023; Bayane, 2026). Formal recognition could enhance police responsiveness, codify operating norms, and strengthen public confidence (Fairwork, 2021). Likewise, drivers’ concerns about oversupply reflect platform-induced market saturation: continuous onboarding reduces trip volumes, depresses earnings, and intensifies competition. Regulation addressing entry and market order, without veering into protectionism, could mitigate destructive competition and reinforce occupational health and safety commitments (Heeks et al., 2021; Fairwork, 2021). Drivers’ framing of oversupply as a platform-driven phenomenon rather than a natural market outcome highlights how digital platform design itself produces economic instability and motivates calls for regulatory control over driver intake.

By centring drivers’ own regulatory preferences within a contested legal and transport environment, this study contributes to sociological debates on algorithmic labour, precarity, and industrial relations in the Global South. It clarifies why many platform workers support partial regulation that preserves temporal autonomy while instituting enforceable protections and identifies context-appropriate policy levers for South Africa’s urban mobility labour market (De Stefano, 2016; Prassl, 2018; Anwar and Graham, 2021; Woodcock and Graham, 2020). These insights resonate strongly with global concerns around Sustainable Development Goal 8 on Decent Work and Sustainable Development Goal 16 on institutional accountability, reinforcing the need for regulation that balances innovation with equity. Finally, an inclusive and participatory policymaking approach, engaging the state, Uber, civil society, and drivers, is essential for designing frameworks that reflect the lived realities of platform work in South Africa and across diverse geographic contexts (Heeks et al., 2021).

## 7 Conclusion

This study demonstrates that Johannesburg Uber drivers’ perspectives on regulation are shaped by the dual pressures of algorithmic control and structural precarity, resulting in a pragmatic yet ambivalent stance that seeks protection without sacrificing flexibility. While drivers support regulation that enhances safety, earnings stability, due-process mechanisms, and market order, they remain wary of formalisation that could erode the limited autonomy afforded by platform work. These preferences reflect broader dynamics of platform labour in Johannesburg, where high unemployment, migrant precarity, and volatile operating conditions intensify insecurity and make hybrid regulatory solutions both desirable and necessary. The study contributes to sociological debates on algorithmic management, worker classification, and industrial relations, highlighting the need for context-sensitive, participatory regulatory frameworks that can balance innovation with fairness and align with decent-work objectives in Johannesburg’s evolving urban mobility sector.

## Data Availability

The data analysed in this study is subject to the following licences/restrictions: data cannot be shared with the public due to confidentiality purposes. Requests to access these datasets should be directed to Percyval Bayane – bayanp@unisa.ac.za.
